# Myocardial Iron Loading Assessment by Automatic Left Ventricle Segmentation with Morphological Operations and Geodesic Active Contour on T2* images

**DOI:** 10.1038/srep12438

**Published:** 2015-07-28

**Authors:** Yun-gang Luo, Jacky KL Ko, Lin Shi, Yuefeng Guan, Linong Li, Jing Qin, Pheng-Ann Heng, Winnie CW Chu, Defeng Wang

**Affiliations:** 1 Department of Stomatology, The Second Hospital of Jilin University, Changchun, 130041, Jilin Province, China; 2 Research Center for Medical Image Computing, Department of Imaging and Interventional Radiology, The Chinese University of Hong Kong, Shatin, New Territories, Hong Kong, China; 3 Department of Medicine and Therapeutics, The Chinese University of Hong Kong, Shatin, NT, Hong Kong SAR; 4 Shenzhen Institutes of Advanced Technology, Chinese Academy of Science, Shenzhen, China; 5 Department of Computer Science and Engineering, The Chinese University of Hong Kong, Shatin, New Territories, Hong Kong, China; 6 CUHK Shenzhen research institute, Shenzhen, China; 7 Department of Biomedical Engineering and Shun Hing Institute of Advanced Engineering, The Chinese University of Hong Kong.

## Abstract

Myocardial iron loading thalassemia patients could be identified using T2* magnetic resonance images (MRI). To quantitatively assess cardiac iron loading, we proposed an effective algorithm to segment aligned free induction decay sequential myocardium images based on morphological operations and geodesic active contour (GAC). Nine patients with thalassemia major were recruited (10 male and 16 female) to undergo a thoracic MRI scan in the short axis view. Free induction decay images were registered for T2* mapping. The GAC were utilized to segment aligned MR images with a robust initialization. Segmented myocardium regions were divided into sectors for a region-based quantification of cardiac iron loading. Our proposed automatic segmentation approach achieve a true positive rate at 84.6% and false positive rate at 53.8%. The area difference between manual and automatic segmentation was 25.5% after 1000 iterations. Results from T2* analysis indicated that regions with intensity lower than 20 ms were suffered from heavy iron loading in thalassemia major patients. The proposed method benefited from abundant edge information of the free induction decay sequential MRI. Experiment results demonstrated that the proposed method is feasible in myocardium segmentation and was clinically applicable to measure myocardium iron loading.

Congestive heart failure is the main cause of death in thalassemia major patients and is traditionally referred as myocardial iron overload^1^,^2^,^3^. Iron chelation therapy is an efficient treatment to remove excessive iron from the human body^4^,^5^,^6^. To treat thalassemia major in its early stage, the myocardium iron loading should be assessed by T2* measurements based on the segmentation results of the endocardium and epicardium^7^,^8^. However, accurate segmentation of the myocardium in cardiac MRI is a labor intensive work for an experienced cardiologist, which limits its applications.

Manual segmentation of the myocardium is subjective depending upon the operators’ knowledge and experience. To overcome such a drawback, various semi-automatic and fully- automatic segmentation techniques were developed to improve the segmentation performance. In 2005, Montagnat and Delingette^9^ extended the deformable surface framework by introducing time-dependent constraints, and it was successfully applied to 4D cardiac image segmentation. McInerney and Terzopoulos proposed a dynamic finite element surface model for segmentation and tracking in multidimensional medical images, and achieved satisfactory performance cardiac 4D image analyses^10^. Recently, the active shape model (ASM) and active appearance model (AAM) were adopted for 2D/3D medical image segmentation^11^,^12^,^13^.

In the maximum likelihood framework, Rousson and Paragios introduced the shape priors into level set representations for 2D image segmentation. The level set representation has been widely used in image processing and computer vision due to its implicit, intrinsic, parameter and topology freedom^14^. To deal with the left ventricle segmentation in cardiac MRI, Paragios *et al.* presented a level-set-based shape model by combining the visual information, anatomical constrains, and a flexible shape-driven cardiac model^15^. Then Charpiat *et al.* proposed a distance function shape representation to further improve the segmentation performance^16^. However, significant challenges restricted the implementation of those techniques to provide a satisfactory performance with respect to the cardiac MRI segmentation. The endocardium boundary between the myocardium and the blood pool was difficult to define, because of the protruding papillary muscles (which should not be ignored), in the cardiac cavity. Poor boundary information prevented the epicardium from being easily distinguished from the cardiac and surrounding tissues in MR images^17^. Cordero-Grande, L. *et al.* suggested an unsupervised 4D myocardium segmentation using a Markov Random Field which obtained results in good agreement with those from experienced cardiologists, but required long computational time^18^.

To further improve the segmentation performance, the level set based active contour model has drawn the attention from a wide range of researchers from different fields. Lynch *et al.* adopted a coupled level-set based active contour model to accurately perform left-ventricle myocardium segmentation^19^. It had two advantages. 1. The evolution contour could change its topology during the level set evolution; 2. The hybrid method with level set and active contour could be easily extended to higher dimensions. But this technique also suffered from a long computational time cost. In this study, we proposed to complete the segmentation process based on the integrated edge information of different signal intensity peak images during T2* data acquisition and to use a geodesic active contour (GAC) method to improve the performance of the myocardium segmentation for iron loading assessment.

According to the segmentation result, the T2* value of the myocardium was calculated to analyze iron loading in patients with thalassemia major. High reproducibility of manual myocardium segmentation was resource demanding for experienced operators. Here, we demonstrate the consistently superior segmentation performance with our fully-automatic process approach.

## Materials and methods

The methods were carried out in accordance with the approved guidelines of *Scientific Reports*.

### Subjects and MRI data acquisition

The study protocol was approved by the Clinical Research Ethics Committee and carried out in accordance with the approved guidelines. Informed written consent was obtained from all subjects. 26 patients (male: 10, female: 16, mean age =22.7 years, s.d. = 7.3 years, range: 10.0–38.1 years) with thalassemia major were recruited.

All cardiovascular MRI examinations were performed on a 1.5T whole body human platform (MEGNETOM Sonata, Siemens Medical System, Erlangen, Germany). Eight-peak spectral model T2* value was evaluated by a cardiac-gated single breath-hold technique. Eight images were collected at tele-diastolic phase in short axis view with repetition time TR = 160 ms, echo times TE = {2.6, 4.6, 6.6, 8.7, 10.7, 12.7, 14.7, 16.7}±0.1 ms, echo train length = 1, flip angle = 20°, and matrix = 144 (left-right) × 256 (anterior-posterior), pixel spacing = 1.5625 mm × 1.5625 mm, number of slices = 1, bandwidth = 815 Hz/pixel. Data were processed using an in-house developed algorithm from MATLAB (version 2012a, The Math Works Inc., Natick, Massachusetts, US).

### Image registration

Cardiac MR Images possessed slight deformations due to heart beating motion. In our research, individual echo time images were aligned to the first echo time image of each scan. The alignment was achieved by using the mutual information (MI) linear registration technique^20^.

### Left ventricle detection

**Figure 1:**
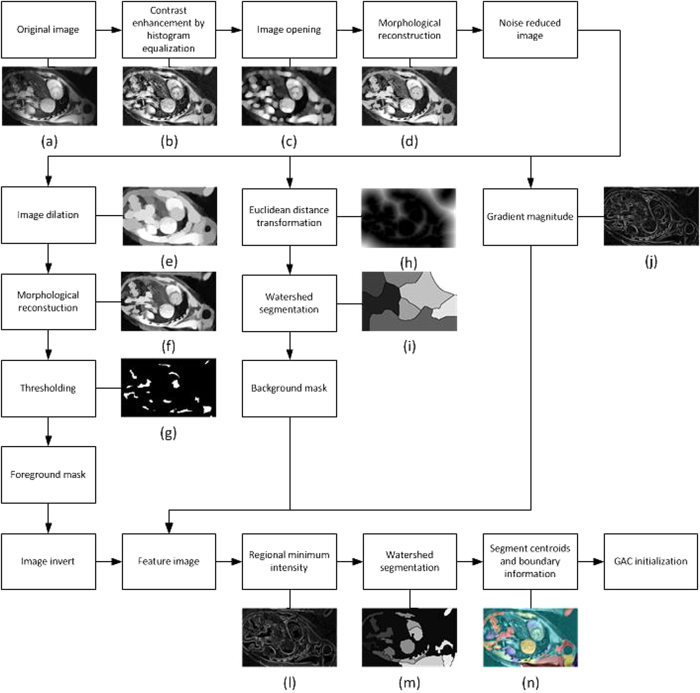
Workflow of automatic left ventricle detection.

The left ventricle in the short axis view possessed two characteristics which allowed for automatic localization. The detection algorithm aimed to search for both hyperintensity and a large circular blood pool in the thoracic MR image. The image with the shortest echo time was chosen for it has the highest contrast among all images within a single scan. Before the feature extraction process, noise reduction procedures were performed with contrast enhancement (Fig. 1b), morphological opening (Fig. 1c) and morphological reconstruction (Fig. 1d).

Feature extraction was achieved by combining foreground masks, background masks, and gradient magnitude images. The foreground mask was defined as a threshold mask (Fig. 1g) of a morphologically reconstructed dilate image (Fig. 1e,f). A watershed segmentation (Fig. 1i)^21^ was applied on the noise reduced image with the Euclidean distance transformation (Fig. 1h)^22^ as the background mask. The feature image was generated by combining the inverted foreground mask, background mask, and gradient magnitude (Fig. 1j) images with regional minimum intensities (Fig. 1l). The watershed algorithm was applied again on the feature image and hence a clear segmentation image resulted (Fig. 1m). Moreover, centroids and boundary-to-centroid information was obtained for individual segments. By excluding segments in contact with image edges and choosing the regions near the image center with average intensity higher than customized threshold value. (Fig. 1n).

### Geodesic active contour (GAC)

The fundamental objective of GAC model was to track a closed surface Γ(*s*), for which Γ(*s*):[0:∞] → *R*^*N*^ as it evolved in data space^23^. Such an interface was represented as a closed curve *C*(*s*) in 2D or a set of points on the boundaries of the region of interest Ω . Initially introduced by Osher and Sethian^24^, the level set (geodesic) method performed well at capturing dynamic interfaces and shapes. The basic idea of this method was that the contour could be embedded as the zero level set of a high-dimensional function *ϕ*(*x*, *y*, *t*) known as a zero level set function (LSF). Assuming a dynamic parametric contour 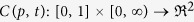 with a spatial parameter *p* ∈ [0, 1] , and *t* was a temporal variable *t* ∈ [0, ∞), then the target contour was described as the zero level set function
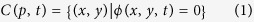


Embedding level set function *ϕ* which was described by the continuous Lipschitz function with signed distance *d* from (*x*, *y*) to the initial curve *C*_0_. Such a Lipschitz function implied that the existence of a bounded first derivative. The distance was given a positive sign outside the initial boundary (*D*Ω), a negative sign inside the boundary (Ω\∂Ω) and zero on the boundary (∂Ω).
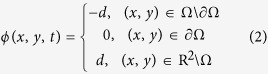


In Riemannian space, length is defined as
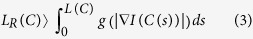
Where *s* denotes the arc length parameter, *g* represents a positive decreasing function, *L*(*C*) is the Euclidean arc length of *C*(*s*) . ∇*I* in equation^8^ is a gradient value measured across a four connected 2D neighborhood. As the Riemannian length *L*_*R*_(*C*) is obtained by weighting the Euclidean length element *ds* by *g*(|∇*I*(*C*(*s*))|) boundary information is intrinsically embedded. However, such a model would stop the curve evolution at any image minima. As a consequence, the GAC model is modified as
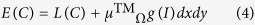
for *μ* is a positive real constant and the second term of equation (4) is considered as an area constraint.

The level set model aims to exchange Lagrangian formalization and replace with an Eulerian partial differential equation with initial values. To minimize the curve for the steepest descent approach, the above equation is equivalent to
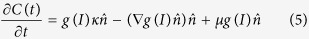
For *κ* is the Euclidean curvature, 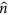 is the unit inward normal vector to the curve *C* . The curvature constant *μ* describes the evolve direction of the active contour curve. For *μ* > 0, the GAC will evolve in the inward direction, or in opposite direction when *μ* < 0.

Expressing the curve evolution with inward normal vector 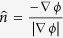 and normal velocity vector 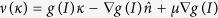, the curve evolution equation (5) was finally expressed as
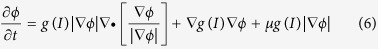
For *I* is one of the eight sequential images, the edge indicator function *g* may be used to control the curve evolution and stop the curve from evolving when it arrives at an object’s boundaries. Such a function is defined to be
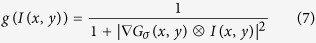
where ⊗ is the convolution operator and 
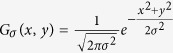
 is a Gaussian kernel with standard deviation *σ* .With this definition, |∇*G*_*σ*_⊗*I*|^2^→0 and *g*(*I*(*x*, *y*))→1 in homogenous regions without fine texture. In contrast, |∇*G*_*σ*_⊗*I*|^2^→255^2^ and *g*(*I*(*x*, *y*))→0 at edge regions.

### Image segmentation base on boundary information

#### Edge detector function

Edge information of the epicardium and endocardium was indistinctive in a single cardiac MR image due to additive/multiplicative noise and the surrounding tissues. To improve the myocardium segmentation result, we proposed to integrate edge information from all eight sequential images. The edge detector function for the endocardium edge was defined as follows:
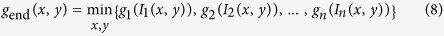
where (*x*, *y*) ∈ image domain, and the edge detector functions of the multi-sequential images *g*_1_, *g*_2_,...*g*_*n*_ can be obtained respectively from Eq. (7). However, the epicardial-myocardial junction is difficult to be ascertained in clinical applications due to the right ventricle, liver, and other complex tissues in neighbouring regions. In order to avoid these negative effects in our segmentation process, the epicardium edge indicator function was defined as
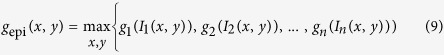


The maximum value of the eight edge detector functions at the same point can be chosen to be the new edge indicator function, which can eliminate the impacts of weak edges. Combining Equation (6), (8) and (9), the initial contours of the endocardium *ϕ*_*end*_ and epicardium *ϕ*_*epi*_ will be described by the curve evolutions
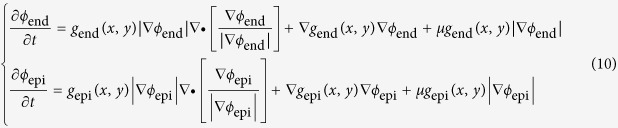


#### GAC initialization and parameter setting

To demonstrate the advantage of the hybrid segmentation method by combining GAC and level sets based on abundant edge information, the eight sequential images underwent image registration with the mutual information method. In the numerical implementation, we set Δ*t* = 0.1, *μ* = 0.5 and *σ* = 1.2 for both images.

Myocardium was assumed to be circular in shape. The centers of initial contours *ϕ*_end_ and *ϕ*_epi_ were defined as the left ventricle centroid found by the automated detection scheme. The initial radius of *ϕ*_epi_ was set as mean distance from centroid to boundary of left ventricle after watershed segmentation in automated detection scheme. Radius of *ϕ*_end_ was 75% of initial radius of *ϕ*_epi_. Manual adjustments were implemented for a false left ventricle centroid identification. The curve evolution was iterated for 1000 times then further analyzed with T2* value.

### T2* analysis

Duo to inhomogeneity of iron deposits, the segmented left ventricle myocardium was further divided into 6 sectors according to the 16 myocardial standard segments^25^,^26^,^27^. The mean intensity value of each sector of the eight sequential images was implemented with a T2* analysis for iron loading assessment. To compute the T2* values in each sector, an exponential trend-line with the fitted equation was used
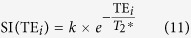
where *k* is a constant, TE_*i*_ is the echo time of the i-th image, and SI represents the image intensity within the sector region.

The T2* relaxation time is inversely proportional to the slopes of the decay curves, thus the higher slope of decay curve, T2* value is a lower value. As discussed in^28^,^29^, the patients were diagnosed to be normal when the cardiac T2* value was more than 20 ms.

## Result

### Automatic left ventricle detection and segmentation result

The proposed method was tested on cardiac MR images from 26 patients with thalassemia major. To maintain the consistency of the results, the parameters were unchanged for all datasets assessed. Correction detection, or known as true positive detection, was defined as one of the detected centroids were lied within left ventricle blood pool. In contrast, false positive detection was defined as all detected centroids were lied outside the left ventricle blood pool. For the algorithm didn’t have a negative output, true negative and false negative was not applicable.

**Figure 2:**
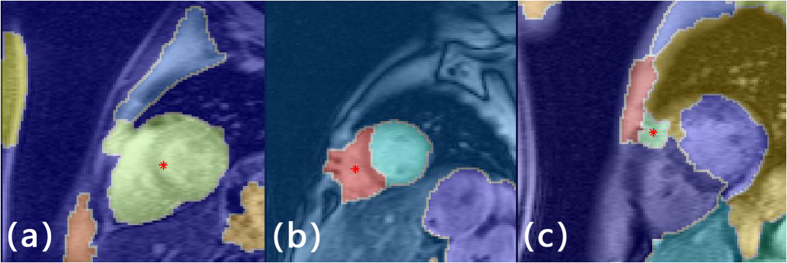
Examples of false left ventricle detection (**a**) Left ventricle and left atrium is consider as one single segment. (**b**) Misidentify left atrium as left ventricle. (**c**) Detected centroid outside heart region.

**Figure 3:**
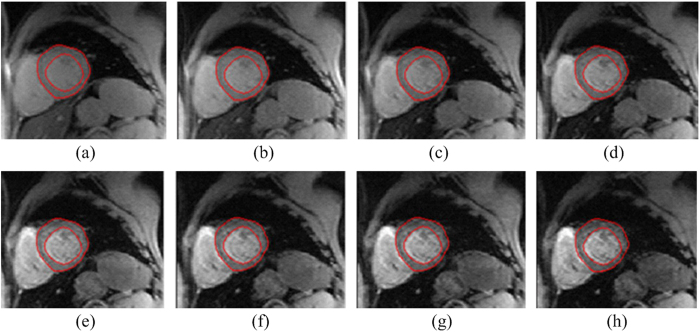
Myocardium segmentation results generated with the registration process are represented by solid red lines. Both the segmented endocardial and epicardial contours have similar geometric features in the sequential cardiac MRI images (**a-h**). This segmentation procedure has the advantage of low computational cost and could enable the effective assessment of myocardium iron loading.

**Figure 4:**
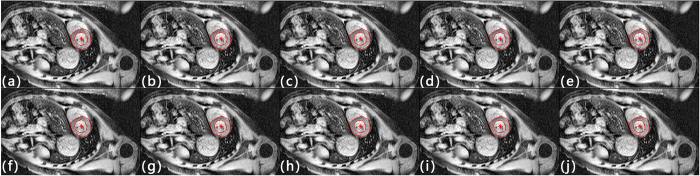
GAC segmentation results with iteration number from 100–1000 (a–j).

Base on this definition, the left ventricle detection scheme has a true positive detection rate of 84.6% and false positive rate of 15.4%. There are three types of false positive detections, 1. Mixing left ventricle and left atrium as a whole (Fig. 2a), 2. Misidentifying left atrium as left ventricle (Fig. 2b), and 3. Detected centroid outside heart region (Fig. 2c). Results from traditional manual and proposed automatic GAC segmentations were compared in terms of areal accuracy and computational speed. The average areal variation and CPU execution time for iterations 100 to 1000 was listed in Table 1. Segmentation results of one randomly selected patient at different echo times and iteration times were listed in Figs 3 and 4 respectively.

**Figure 5:**
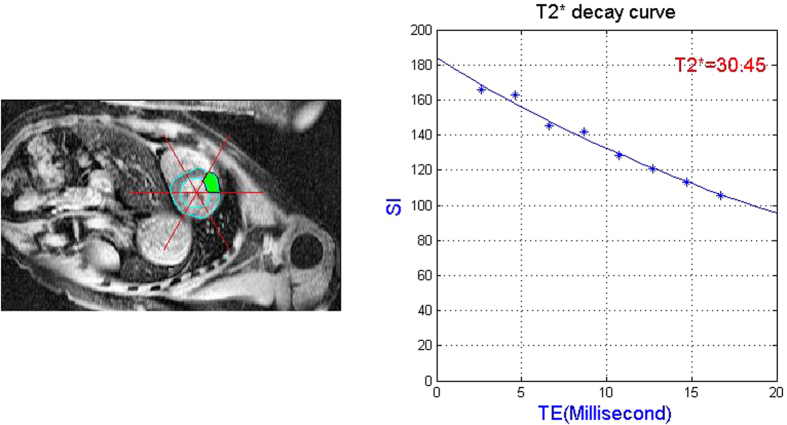
T2* curve fitting on one sector of myocardium. Green labeled region: anterior segment.

Myocardial iron loading was assessed based on our myocardium segmentation results. The segmented myocardium was divided equally between six sectors with equal an angular size base on the 16 myocardial standard segments (Fig. 5). Measured T2* results by manual and automatic segmentation method of different patients were listed in Table 2. Mean T2* value of manual and automatic segmentation were whole myocardium region based and sectored region based respectively. Patients were diagnosed to have myocardial iron over-loading when the average cardiac T2* value was less than 20 ms. As the infracted tissues may only occupy part of the myocardium, T2* values varied among different sectors of the same scan. Instead of considering the segmentation region as a whole, a sectored diagnosis provided a more detailed approach in detecting abnormal parts of myocardium.

## Discussion

Automatic organ detection was a challenging task for computer vision. The proposed left ventricle detection has a correct detection rate of 84.6%. False detections were mainly classified as (a) left ventricle and left atrium was detected as one segment, (b) the algorithm cannot distinguish left ventricle and left atrium. Possible reason of first type misidentification was caused by high image intensity at anterospetal myocardium region due to high iron accumulation. Low contrast between the blood pools and the myocardium thus form a low local image gradient. The gradient magnitude, however, was one major element in the automatic detection scheme. A superior contrast enhancement algorithm was suggested to replace the histogram normalization method. More research should be conducted in order to find the optimal contrast adjustment method.

The proposed left ventricle detection method aimed to search for large, circular and hyperintensive blood pool in the image. However, both left ventricle and left atrium possessed similar morphological features as the left ventricle to the short axis view cardiac MRI. One solution suggested to use areal constrain to classify left atrium and left ventricle, but the hypothesis required more researches to support.

Moreover, myocardium segmentation was composited by the distinct epicardium and endocardium boundary contours. Other tissue, however, usually has only one boundary. This aspect provides a clue to reduce the false positive rate in a future left ventricle detection programme.

**Figure 6:**
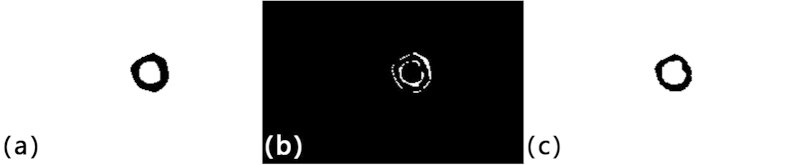
(**a**) Automatic segmentation mask. (**b**) Difference in automated and manual segmentation. (**c**) Manual segmentation mask.

The variation between the manual and automatic segmentation areas is about 25% for all iterations. By visual examination, the GAC segmentations generally have a larger endocardium boundary than the manual ones (Fig. 6). Since the automatic segmentation programme adopted the edge detector function with eight echo time images, boundary information was more abundant than with manual segmentation on a single echo time slice. The boundary between left ventricle and surrounding tissues may not be clearly defined in a single echo time image. During the free induction decay process, the differing relaxation rates of various tissues, create an image contrast. The newly adopted edge-detecting function, was then enhanced by jointly using the images from different echo times. Benefiting from the abundant boundary information of the multi-echo images, the epi- and endocardium were better defined.

The iteration process was sensitive to the papillary muscles, which were excluded in the manual segmentations. Although fine adjustment of GAC parameters can improve the endocardium segmentation results, this was impractical in a large data analysis. Sequence parameters would also affect the segmentation results as adaptation of higher resolution and contrast images would provide finer edge details.

The computational speed of the GAC iteration was slower than the manual segmentations. The CPU execution speed depended upon the hardware and software programming. In comparison with traditional labor-intensive and experience-depending manual segmentation, the newly developed model can operate without any prior anatomical knowledge. The advantage of using the GAC as a segmentation algorithm is the high level of robustness for initializing a contour, given that the longer computation time in return. Also the proposed algorithm was more objective and reproducible. The major process which slowed down the proposed algorithm was the re-initialization of an active contour, which spent 5.5 seconds for every 100 iterations on average. Comparing the average area difference between manual and automatic segmentation, it was found that the result did not improve much even more iterations were applied. The development of a faster and more accurate programme would be the major focus of work in the future.

In comparison with existing automatic/semi-automatic myocardium segmentation method, the proposed scheme has shown its superiority in terms of computational speed and robustness. Unsupervised random Markov field method has obtained results with good agreement with cardiologists^18^. However, the method was computational demanding (~6 min for sever and ~56 min for PC). In contrast with 1 minute computational cost for the proposed method by using a typical desktop PC, the proposed method was clearly more clinical applicable.

Another advantage of the proposed scheme was the unsupervised algorithm, while most other artificial intelligent segmentation methods required a machine training process^12^,^19^,^30^,^31^. Segmentations result of those supervised methods were highly depending on the training data. In clinical environment, pre-trained models may not fit abnormal cardiac images. Robustness of the unsupervised scheme was believed to be clinically more applicable than supervised one. More researches should be conducted to give a solid statistical support in the future.

The correlation between myocardial T2* and transfusion duration has revealed an increased risk of cardiac symptoms^32^. The inhomogeneous distribution of iron deposits^33^ is accessible through our six sectored T2* analysis. In comparison with a cardiac biopsy, MR imaging allows a safe, whole heart assessments, showing a significant advantage compared to pathological examination.

## Conclusion

The current cardiac iron loading assessments by MR images is labor intensive and operator dependent. We propose a fully automatic left ventricle segmentation process to improve the efficiency of myocardium iron loading assessment. Instead of detecting the organ boundary with a single slice, the recently developed method was based on multi-echo sequential images. Though several technical issues remain to be solved, the proposed method was a pioneering research in radiology imaging for organ detection and segmentation. Cardiac iron loading assessment can now achieved without being dependent upon an operator’s prior knowledge of anatomical details.

## Additional Information

**How to cite this article**: Luo, Y.- *et al.* Myocardial Iron Loading Assessment by Automatic Left Ventricle Segmentation with Morphological Operations and Geodesic Active Contour on T2* images. *Sci. Rep.* **5**, 12438; doi: 10.1038/srep12438 (2015).

## Figures and Tables

**Table 1:** Average computational time in 100 iteration steps and area difference in comparison with manual segmentation

**Iteration number**	**100**	**200**	**300**	**400**	**500**	**600**	**700**	**800**	**900**	**1000**
Average execution time for auto left ventricle detection (seconds)	28.1	34.7	41.3	43.5	49.3	53.8	60.3	65.9	73.9	77.5
Average area difference in comparison with manual segmentation (%)	25.5	25.7	25.5	25.1	25.1	26.0	26.8	25.7	25.5	25.5

**Table 2:** T2* measurement result of segmented myocardium.

**Patient**	**Sector 1 (ms)**	**Sector 2 (ms)**	**Sector 3 (ms)**	**Sector 4 (ms)**	**Sector 5 (ms)**	**Sector 6 (ms)**	**Average (ms)**	Clinical Liver T2* (ms)	Clinical Heart T2* (ms)	**Remark**
1	32.10	22.78	37.92	41.67	33.71	33.71	33.65	>6.3	>20	
2	41.03	41.09	65.12	65.62	32.49	32.49	46.31	NA	NA	
3	23.78	21.31	19.32	19.92	19.56	19.56	20.58	1.44	22.07	
4	40.16	35.34	62.10	62.34	32.49	32.49	44.16	NA	NA	
5	41.44	36.85	54.12	56.58	36.67	36.67	43.72	NA	NA	
6	25.54	24.94	37.05	27.64	31.72	31.72	29.77	1.89	33.61	
7	19.43	17.49	20.20	20.97	20.06	20.06	19.70	1.01	16.92	
8	30.07	29.87	36.89	57.10	25.90	25.90	34.29	NA	NA	
9	27.43	16.69	14.88	15.83	15.24	15.24	17.55	NA	NA	
10	20.93	21.35	20.46	14.13	13.07	13.07	17.17	NA	NA	
11	20.68	18.99	55.68	60.00	41.75	41.75	39.81	1.20	56.60	On iron chelation
12	35.81	33.44	33.38	81.16	60.96	60.96	50.95	1.23	61.62	
13	11.84	8.62	12.34	17.31	16.94	16.94	14.00	2.30	9.03	
14	24.60	21.46	22.25	42.16	72.21	72.21	42.48	10.56	43.63	
15	40.12	25.97	47.66	60.39	34.66	34.66	40.58	2.69	49.23	On hydroxyurea
16	29.44	31.80	40.41	46.96	29.28	29.28	34.53	4.22	41.19	Post-splenectomy
17	40.18	70.40	41.37	57.82	25.68	25.68	43.52	2.05	45.70	High serum ferritin
18	38.94	30.85	41.07	53.22	37.79	37.79	39.94	2.25	41.13	
19	54.51	39.40	60.06	56.37	39.57	39.57	48.24	3.14	53.02	
20	59.43	60.29	81.39	52.51	29.50	29.50	52.10	5.93	38.22	Extramedullary hematopoiesis
21	52.46	44.53	43.95	42.48	34.78	34.78	42.16	6.38	34.74	
22	22.82	22.61	26.63	26.80	18.14	18.14	22.52	1.68	21.22	High serum ferritin
23	33.62	21.26	45.96	59.33	33.79	33.79	37.96	3.20	40.94	
24	31.76	13.02	17.06	16.52	15.18	15.18	18.12	7.40	12.39	on iron chelation
25	41.52	64.59	40.17	53.22	70.27	70.27	56.67	4.88	58.26	
26	30.59	24.24	34.79	23.43	21.54	21.54	26.02	2.17	22.54	

Clinical results were measured by experienced radiologist with manual segmentation. Sector 1: Anterior, Sector 2: Anterolateral, Sector 3: Inferolateral, Sector 4: Inferior, Sector 5: Inferoseptal, Sector 6: Anteroseptal.
